# Low dose radiation and circulatory diseases: a brief narrative review

**DOI:** 10.1186/s40959-015-0007-6

**Published:** 2015-11-26

**Authors:** Mark P. Little, Steven E. Lipshultz

**Affiliations:** 1 Radiation Epidemiology Branch, National Cancer Institute, Bethesda, MD 20892-9778 USA; 2 Department of Pediatrics, Wayne State University School of Medicine and Children’s Hospital of Michigan, Detroit, MI 48201-2196 USA; 3 National Cancer Institute, Room 7E546, 9609 Medical Center Drive, MSC 9778, Rockville, MD 20892-9778 USA

**Keywords:** Circulatory disease, Radiation, Heart disease, Stroke, Review

## Abstract

Exposure to high doses of ionizing radiation is associated with damage to the heart and coronary arteries. However, only recently have studies with high-quality individual dosimetry data allowed this risk to be estimated while adjusting for concomitant chemotherapy. An association between lower dose exposures and late-occurring circulatory disease has only recently been suspected in the Japanese atomic bomb survivors and in various occupationally exposed cohorts and is still controversial. Excess relative risks per unit dose in moderate- and low-dose epidemiological studies are variable, possibly resulting from confounding and effect-modification by well known (but unobserved) risk factors. Here, we summarize the evidence for a causal association between moderate- and low-level radiation exposure (whether at high or low dose rates) and circulatory disease.

## Background

Circulatory disease is the leading cause of death in the developed world [1]. There are many types of circulatory disease [2]. Consistently identified independent risk factors include cigarette smoking, diabetes, high blood pressure, obesity, and increased total and low-density-lipoprotein cholesterol [3]. Of emerging importance are certain maternal reproductive factors [4, 5].

The health risks of low-level exposure to ionizing radiation are usually thought to be related primarily to cancer in the directly-exposed population [6, 7]. Most cancer types have been associated with radiation exposure, whether in the Japanese atomic bomb survivors [8, 9] or in other groups [6]. At high radiation doses (>5 Gy), a variety of other well established effects are observed; in particular, damage to the structures of the heart and to the coronary, carotid, and other large arteries [10]. Several recent reviews have suggested an excess radiation-induced risk at occupational and environmental dose levels (<0.5 Gy 1) [2, 11–15], although the presence and magnitude of low-dose risk is still unclear, with some advocating for the use of a threshold [16, 17]. The results of a recent meta-analysis suggest that if the risks were applied to various current populations that overall radiation-related mortality would be about twice that currently estimated based on estimates for cancer endpoints alone [13].

Here, we briefly summarize the evidence, including that from several systematic reviews [12-15], for a causal association between moderate- and low-level radiation exposure (whether at high or low dose rates) and cardiovascular disease because of its potential impact on radiation detriment.

## Review

Damage to the vasculature can affect the function of most body organs by restricting blood flow and oxygen to tissue; however, the heart and brain are of main concern. At high radiation doses (organ doses > 5 Gy), such as those received by patients treated with radiotherapy, a variety of adverse effects in the circulatory system have been reported, among them damage to the structures of the heart—including marked diffuse fibrotic damage, especially of the pericardium and myocardium, pericardial adhesions, microvascular damage, intracardiac conduction system, and stenosis of the valves—and to the coronary, carotid, and other large arteries. These effects occur both in patients receiving radiotherapy and in experimental animals [10]. There are plausible, if not completely understood, inflammatory mechanisms by which high-dose radiation might affect the blood-circulatory system [18].

### Findings in the Japanese atomic bomb survivors

Excess radiation-associated mortality from heart disease and stroke has been observed in the Life Span Study (LSS) cohort (Table 1) [19]. In the latest follow-up of the Adult Health Study (AHS), a subset of the LSS cohort, Yamada et al. [20] observed generally non-statistically significant, radiation-associated excess risks of hypertension and myocardial infarction (Table 1). Analysis within the AHS of those exposed in early childhood showed a significantly increased incidence of non-fatal stroke or myocardial infarction, although there was no excess risk among those exposed in utero for whom the average exposures were much lower [21] (Table 1). The studies of Yamada et al. [20] and Tatsukawa et al. [21] were the only epidemiological reports, apart from those of Ivanov et al. [22] and Azizova et al. [23, 24], that assessed morbidity rather than mortality.

**Table 1:** Estimated excess relative risks of circulatory disease in various studies of moderate- and low-dose radiation exposure. (Adapted from Little et al. [11, 12]). All data are in relation to underlying cause of death, unless otherwise indicated

Cohort/Study	Reference	Mean (range) [SD] heart/brain dose, Sv	Cohort, n (person years of follow-up)	Endpoint (mortality unless otherwise indicated)	Excess relative risk Sv^−1^ (95 % CI)
Japanese atomic bomb survivors					
Japanese atomic bomb survivors	Shimizu et al. [19]	0.1 (0–4)^a^	86,611 (n.a.)	Ischemic heart disease (ICD9 410–414)	0.02 (−0.10, 0.15)
				Myocardial infarction (ICD9 410)	0.00 (−0.15, 0.18)
				Hypertensive heart disease (ICD9 402, 404)	0.37 (0.08, 0.72)
				Rheumatic heart disease (ICD9 393–398)	0.86 (0.25, 1.72)
				Heart failure (ICD9 428)	0.22 (0.07, 0.39)
				Other heart disease (ICD9 390–392, 415–427, 429)	−0.01 (−0.21, 0.24)
				Hypertensive disease without heart disease (ICD9 401, 403, 405)	0.07 (−0.22, 0.55)
				Heart disease total (ICD9 393–429 excluding 401, 403, 405)	0.18 (0.11, 0.25)^b^
				Cerebral infarction (ICD9 433,434)	0.04 (−0.10, 0.20)
				Cerebral hemorrhage (ICD9 431)	0.05 (−0.06, 0.17)
				Subarachnoid hemorrhage (ICD9 430)	0.30 (−0.04, 0.76)
				Other or unspecified cerebrovascular disease	0.16 (0.01, 0.34)
				Cerebrovascular disease total (ICD9 430–438)	0.12 (0.05, 0.19)^b^
				Circulatory disease apart from heart disease and stroke (ICD9 390–392, 401, 403, 405, 439–459)	0.58 (0.45, 0.72)^b^
				Other circulatory disease (ICD9 399–400, 406–409, 439–459)	−0.01 (<−0.01, 0.34)
				All circulatory disease (ICD9 390–459)	0.15 (0.10, 0.20)^b^
Japanese atomic bomb survivors	Yamada et al. [20]	0.1 (0–4)^b^	10,339 (n.a.)	Hypertension incidence, 1958–1998 (ICD9 401)	0.05 (−0.01, 0.10)^c^
				Hypertensive heart disease incidence, 1958–1998 (ICD9 402, 404)	−0.01 (−0.09, 0.09)^c^
				Ischemic heart disease incidence, 1958–1998 (ICD9 410–414)	0.05 (−0.05, 0.16)^c^
				Myocardial infarction incidence, 1964–1998 (ICD9 410)	0.12 (−0.16, 0.60)^c^
				Occlusion incidence, 1958–1998 (ICD9 433, 434)	0.06 (−0.11, 0.30)^c^
				Aortic aneurysm incidence, 1958–1998 (ICD9 441, 442)	0.02 (−0.22, 0.41)^c^
				Stroke incidence, 1958–1998 (ICD9 430, 431, 433, 434, 436)	0.07 (−0.08, 0.24)^c^
Japanese atomic bomb survivors	Tatsukawa et al. [21]	0.001 (0–1.79)	506 (9265)	Morbidity in utero: hypertension	0.20 (−0.39, 1.38)
				Morbidity in utero: nonfatal stroke or myocardial infarction	−0.91 (−1.00, 79.3)
Japanese atomic bomb survivors	Tatsukawa et al. [21]	0.13 (0–3.53)	1053 (20,216)	Morbidity: hypertension	0.15 (−0.01, 0.34)
				Morbidity: stroke or myocardial infarction	0.72 (0.24, 1.40)
Occupational studies					
Mayak workers	Moseeva et al. [24]	0.62 ± 0.80 (males)^d^	18,856 (356,880)	Ischemic heart disease morbidity (ICD9 410–414)	0.160 (0.089, 0.230)^d, e^
	Azizova et al. [23]	0.51 ± 0.68 (females)^d^	22,377 (425,735)	Cerebrovascular disease morbidity (ICD9 430–438)	0.49 (0.39, 0.60)^d, e^
Chernobyl emergency workers	Ivanov et al. [22]	0.109 (0 - >0.5)	61,017 (n.a.)	Hypertension (ICD10 I10-I15) morbidity	0.26 (−0.04, 0.56)
				Essential hypertension (ICD10 I10) morbidity	0.36 (0.005, 0.71)
				Hypertensive heart disease (ICD10 I11) morbidity	0.04 (−0.36, 0.44)
				Ischemic heart disease (ICD10 I20-I25) morbidity	0.41 (0.05, 0.78)
				Acute myocardial infarction (ICD10 I21) morbidity	0.19 (−0.99, 1.37)
				Other acute ischemic heart disease (ICD10 I24) morbidity	0.82 (−0.62, 2.26)
				Angina pectoris (ICD10 I20) morbidity	0.26 (−0.19, 0.71)
				Chronic ischemic heart disease (ICD10 I25) morbidity	0.20 (−0.23, 0.63)
				Other heart disease (ICD10 I30-I52) morbidity	−0.26 (−0.81, 0.28)
				Cerebrovascular disease (ICD10 I60-I69) morbidity	0.45 (0.11, 0.80)
				Morbidity from diseases of arteries, arterioles and capillaries (ICD10 I70-I79)	0.47 (−0.15, 1.09)
				Morbidity from diseases of veins, lymphatic vessels and lymph nodes (ICD10 I80-I89)	−0.26 (−0.70, 0.18)
				All circulatory disease (ICD10 I00-I99) morbidity	0.18 (−0.03, 0.39)
German uranium miner study	Kreuzer et al. [31]	0.041 (0–0.909)^d^	58,982 (2,180,639)	All circulatory disease (ICD10 I00-I99)	−0.13 (−0.38, 0.12)^d^
				Ischemic heart disease (ICD10 I20-I25)	−0.03 (−0.38, 0.32)^d^
				Cerebrovascular disease (ICD10 I60-I69)	0.44 (−0.16, 1.04)^d^
Électricité de France workers	Laurent et al. [33]	0.0215 (0–0.6)	22,393 (440,984)	Ischemic heart disease	4.1 (−2.9, 13.7)^f^
				Cerebrovascular disease	17.4 (0.2, 43.9)^f^
				All circulatory disease	2.7 (−2.3, 9.1)^f^
Eldorado uranium miners and processing (male) workers	Lane et al. [32]	0.0522 (<0.0234– > 0.1215)	16,236 (508,673)	Ischemic heart disease	0.15 (−0.14, 0.58)
				Stroke	−0.29 (<−0.29, 0.27)
				All other circulatory disease	0.07 (<−0.33, 0.77)
British Nuclear Fuels plc. workers	McGeoghegan et al. [34]	0.0569 (0– > 0.729)	38,779 (1,081,570)	Ischemic heart disease (ICD9 410–414)	0.70 (0.37, 1.07)^b, f^
				Cerebrovascular disease (ICD9 430–438)	0.66 (0.17, 1.27)^b, f^
				Other circulatory diseases (ICD9 390–398, 415–429, 440–459)	0.83 (−0.10, 1.12)^f^
				Circulatory diseases apart from cerebrovascular (ICD9 390–429, 439–459)	0.72 (0.39, 1.10)^f^
				All circulatory disease (ICD9 390–459)	0.54 (0.30, 0.82)^b, f^
3^rd^ Analysis of UK National Registry for Radiation Workers	Muirhead et al. [29]	0.0249 (<0.01– > 0.4)	174,541 (3.9 × 10^6^)	All circulatory disease (ICD9 390–459)	0.251 (−0.01, 0.54)
				Circulatory disease not strongly related to smoking (ICD9 390–409, 415–440, 442–459)	0.280 (−0.19, 0.85)
				Aortic aneurysm (ICD9 441)	−0.132 (−1.29, 1.92)
				Ischemic heart disease (ICD9 410–414)	0.259 (−0.05, 0.61)
				Cerebrovascular disease (ICD9 430–438)	0.161 (−0.42, 0.91)
US Oak Ridge workers	Richardson and Wing [63]	n.a. (0– > 0.1)	14,095 (425,486)	Ischemic heart disease (ICD8 410–414)	−2.86 (−6.90, 1.18)
International Agency for Research on Cancer15-country nuclear worker study	Vrijheid et al. [27]	0.0207 (0.0– > 0.5)	275,312 (4,067,861)	Circulatory disease (ICD10 I00-I99, J60-J69, O88.2, R00-R02, R57)	0.09 (−0.43, 0.70)
				Ischemic heart disease (ICD10 I20-I25)	−0.01 (−0.59, 0.69)
				Heart failure (ICD10 I50)	−0.03 (<0, 4.91)
				Deep vein thrombosis and pulmonary embolism (ICD10 I26, I80, I82, O88.2)	−0.95 (−1.00, 9.09)^g^
				Cerebrovascular disease (ICD10 I60-I69)	0.88 (−0.67, 3.16)
				All other circulatory disease (ICD10 R00-R02, R57, I00-I99 excluding I20-26, I50, I60-69, I80, I82)	0.29 (<0, 2.40)
Environmental studies					
Three Mile Island study	Talbott et al. [64]	0.0001 (0– > 0.00016)	32,135 (561,063)	Heart disease (white males)	−274 (−874, 438)
				Heart disease (white females)	−951 (−1433, −390)
Techa River study	Krestinina et al. [35]	0.035 (0–0.51)^h^	29,735 (901,563)	All circulatory disease mortality (ICD9 390–459) (10 year lag)	0.24 (−0.08, 0.59)
				All circulatory disease mortality (ICD9 390–459) (15 year lag)	0.36 (0.02, 0.75)
				Ischemic heart disease mortality (ICD9 410–414) (10 year lag)	0.40 (−0.11, 0.99)
				Ischemic heart disease mortality (ICD9 410–414) (15 year lag)	0.56 (0.01, 1.19)
Semipalatinsk nuclear test study	Grosche et al. [36]	0.09 (0–0.63)^d^	19,545 (582,656)	Heart disease (ICD9 410–429): all settlements	3.22 (2.33, 4.10)^d^
				Heart disease (ICD9 410–429): exposed settlements	0.06 (−0.39, 0.52)^d^
				Stroke (ICD9 430–438): all settlements	2.96 (1.77, 4.14)^d^
				Stroke (ICD9 430–438): exposed settlements	−0.06 (−0.65, 0.54)^d^
				Cardiovascular disease (ICD9 390–459): all settlements	3.15 (2.48, 3.81)^d^
				Cardiovascular disease (ICD9 390–459): exposed settlements	0.02 (−0.32, 0.37)^d^

*CI* Confidence Interval, *ICD* International Classification of Diseases

^a^Analysis based on colon dose

^b^Analysis using underlying or contributing cause of death

^c^ Analysis based on stomach dose, derived from Table 4 of Yamada et al. [20] with smoking and drinking in the stratification

^d^Risk estimates in relation to cumulative whole body external gamma dose; doses given here are from Moseeva et al. [24]

^e^Assuming a lag period of 10 years

^f^90 % CI

^g^Estimate derived from log-linear model, evaluated at 1 Sv

^h^Analysis based on dose to muscle

Some aspects of the Japanese atomic bomb survivor data imply that risks may not necessarily apply to other exposed populations. The Japanese atomic bomb survivors, almost uniquely among the groups considered here, were exposed at a high dose rate. Survivors suffered from burns, epilation, and other acute injuries caused by the radiation, heat, and blast of the bombs, respectively, and these injuries, in addition to radiation, may have contributed to the development of non-cancer diseases in later life. In addition to the direct effect of the injuries, these and other trauma might introduce selection bias. Evidence of such bias has been presented by Stewart and Kneale [25], who documented the heterogeneity of risk for various endpoints, in particular cardiovascular disease mortality, among the various acute-injury groups. However, Stewart and Kneale [25] did not consider the effects of dose error. Analysis considering this error provided much reduced and generally not statistically significant evidence for a differential effect among those survivors, especially for cardiovascular disease [26]. Although selection bias cannot be entirely discounted, the general consistency of risks in the Japanese and other groups suggests that it does not have a major impact (Table 1). (For a more formal analysis see reference [13].)

### Occupationally exposed groups

The International Agency for Research on Cancer 15-country study of radiation workers found increasing dose-related trends for mortality from all circulatory disease, cerebrovascular disease, and other circulatory diseases and decreasing trends for ischemic heart disease (IHD), heart failure, deep vein thrombosis, and pulmonary embolism [27] (Table 1), although none of these trends was statistically significant (1-sided *P* ≥ 0.20).

Radiation-associated excess IHD and stroke morbidity were observed in Chernobyl recovery workers, although morbidity from hypertensive heart disease and other heart disease was not increased [22] (Table 1).

A highly statistically significant trend with dose was seen for IHD and cerebrovascular disease in the latest analysis of circulatory disease morbidity and mortality in the Mayak workers [23, 24]. The study is unusual in that doses to certain internal organs, especially the lung and liver, were dominated by doses from internally deposited radionuclides; in particular, the α-particle-emitting radioisotopes of plutonium. Doses in this study are among the highest considered here and arguably were sufficiently high that this study should be considered outside the scope of this review: average whole body doses for external γ rays were 0.5 to 0.6 Gy. However, unlike the partial-body doses received from radiotherapy, the external whole-body doses received by the Mayak workers generally accumulated over a long time, so it is reasonable to include this population here.

Nonetheless, interpreting the results of the Mayak cohort is complicated by the large and highly heterogeneous internal α-particle dose from plutonium. The dose response was significant, both in relation to the external γ dose and the internal (α-particle) dose to the liver [23, 24]. Apart from these workers, few cohorts with α-particle liver dose have individual organ dose estimates, or are large enough to merit analysis of this endpoint. For example, individual α-particle liver dose was generally not evaluated in persons exposed to the diagnostic contrast medium Thorotrast [28].

In the latest analysis of the United Kingdom National Registry for Radiation Workers [29], circulatory disease mortality had a borderline significant trend with dose, with an excess relative risk (ERR) of 0.25 Sv^−1^ (95 % CI, −0.01 to 0.54). In most other workforces [30–33], there were generally no statistically significant trends of circulatory disease with dose (Table 1). Some of these studies overlap and, in particular, substantial portions of the study populations of Muirhead et al. [29] are included in the International Agency for Research on Cancer study [27]. The highly significant excess risks of circulatory disease in a study of British Nuclear Fuels plc workers should also be noted [34]; however, this study is largely subsumed within the study by Muirhead et al. [29] and has only 4 more years of follow-up (to December 31, 2005 versus December 31, 2001 for Muirhead et al. [29]).

A study of a cohort of environmentally exposed individuals in the Southern Ural Mountains reported a statistically significant, or borderline significant, increase (depending on the latent period used) of both all circulatory disease mortality, with an ERR of 0.24 Gy^−1^ (95 % CI, −0.08 to 0.59), and IHD mortality, with an ERR of 0.40 Gy^−1^ (95 % CI, −0.11 to 0.99) with a 10-year lag [35]. The trends were statistically significant (*P* ≤ 0.05) with lags of 15 to 20 years, but not significant (*P* > 0.1) with lags of 0 to 10 years [35].

Grosche et al. [36] studied circulatory disease mortality in a Kazakhstan group exposed to fallout from nuclear weapons tests at the Semipalatinsk site. No excess circulatory disease risk was reported in the group of exposed settlements, with an ERR of 0.02 Gy^−1^ (95 % CI, −0.32 to 0.37) for cardiovascular disease, an ERR of 0.06 Gy^−1^ (95 % CI, −0.39 to 0.52) for heart disease, and an ERR of −0.06 Gy^−1^ (95 % CI, −0.65 to 0.54) for stroke. On the other hand, if exposed and unexposed settlements were analyzed together, the excess risks were highly statistically significant and implausibly large, an ERR of 3.15 Gy^−1^ (95 % CI, 2.48 to 3.81) for circulatory disease, an ERR of 3.22 Gy^−1^ (95 % CI, 2.33 to 4.10) for heart disease, and an ERR of 2.96 Gy^−1^ (95 % CI, 1.77 to 4.14) for stroke. The dosimetry in this cohort is problematic because it is based on assessments of residence, estimates of time spent outdoors, and diet, all of which were collected by interviews more than 30 years after the bomb tests. As such, the results of this study are largely uninformative.

Radiation-associated excess relative risk for circulatory disease does not substantially vary by sex, time since exposure, or age at exposure in Japanese atomic bomb survivors [37, 38], although there are borderline significant decreasing trends with attained age [13, 19]. Increasing time trends have been observed in other groups [27].

## Discussion

Most of the studies considered here involved low to moderate average cumulative radiation doses (0.2 Gy or less), with participants in the occupational studies exposed at near-background dose rates. Nevertheless, the small numbers of participants exposed at high cumulative doses (0.5 Gy or above) drive the observed trends in most cohorts with these higher dose groups (see Table 1).

A particular limitation of many studies considered here is the absence of information on lifestyle, medical and other covariates linked with risk of circulatory disease. Of the studies considered only those of the Japanese atomic bomb survivors [19] and Mayak workers [23, 24] had information on lifestyle factors, in particular cigarette smoking, alcohol consumption, obesity and (in the LSS) a few other variables associated with circulatory disease (diabetes mellitus, education, household occupation). The substantial heterogeneity that was observed for cerebrovascular disease and circulatory disease apart from heart disease and cerebrovascular disease in the previous meta-analysis [13] may not be surprising given the variation in the distributions of different risk factors across populations, but it limits interpretation of the observed associations for these endpoints. However, in most radiation-exposed groups there is little or no evidence that lifestyle factors, when available, interact with radiation-associated circulatory disease risk [19, 23, 24, 39].

There have been a number of recent reviews of candidate biological mechanisms [2, 11, 18]. At high radio-therapeutic doses (>5 Gy), the cell-killing effect on capillaries and endothelial cells plausibly explains effects on the circulatory system [18]. At lower doses (0.5–5 Gy), in humans and in experimental studies, many inflammatory markers are up-regulated long after exposure to radiation. However, for exposures less than about 0.5 Gy, the balance shifts toward anti-inflammatory effects [11, 40]. Interestingly, there is evidence of a steeper dose–response slope for various types of circulatory disease under 0.5 Gy in the US radiologic technologists [41], as also in two groups given highly fractionated fluoroscopic X-ray exposures [42, 43].

The generally uniform whole-body, low linear energy transfer radiation in the cohorts we assessed is uninformative as to specific target tissues. What the target tissues are for circulatory system effects at moderate and low doses (<0.5 Gy) remains uncertain. Dose-related variations in T-cell and B-cell populations in Japanese atomic bomb survivors suggest that the immune system may be adversely affected [44]. There is at best conflicting evidence for involvement of the immune system in cardiovascular disease [45–49]; to the extent that it might be, whole-body or bone-marrow dose could be the most relevant to radiation effects. A mechanism based on monocyte cell killing in the arterial intima suggests that the target for atherosclerosis is the arterial intima [50]; however, this mechanism remains speculative. There is nevertheless suggestive evidence for radiation-induced endothelial cell senescence and associated monocyte adhesion [51]. There is support for the kidney being the target tissue in relation to hypertension in the LSS cohort [52] and in experimental animal data [53].

Diabetes and obesity are major risk factors for circulatory disease [3], the former suggesting that the pancreas may be an etiologically relevant target tissue. Many of the metabolic derangements known to occur in diabetes, including hyperglycemia, excess free fatty acid liberation, and insulin resistance, mediate abnormalities in endothelial cell function [54]. Endothelial cells, because of their strategic anatomic position between the circulating blood and the vessel wall, regulate vascular function and structure; dysfunctions in endothelial cells are thought to be a critical initiating stage in many types of circulatory disease [54]. It has generally been assumed that the heart is the most etiologically relevant target tissue for IHD, and the dose to the heart is often used in analysis of radiation-induced IHD [39, 55]. The critical role of vascular endothelial cells in circulatory disease discussed above suggests that the large arteries (e.g., aorta, carotid), may also be an etiologically relevant target. In the peptic ulcer disease cohort there is a highly significant excess risk of IHD, and a borderline significant excess of cerebrovascular disease [56]. Little et al. [56] considered risk of these endpoints in relation to doses to a number of candidate tissues, including the heart, kidney, thyroid (a surrogate for the carotid artery), pancreas (a surrogate for liver, important also for diabetes) and brain; in relation at least to IHD, the kidney appeared to be etiologically plausible, given the similarity of risk estimates based on kidney-dose with those derived from uniform whole body irradiation in other cohorts, in particular the LSS and various other uniform-whole-body exposed datasets [13]. There is other evidence suggesting a role for radiation therapy, and specifically dose to the pancreas, in causing diabetes, both in the peptic ulcer disease cohort [57] and elsewhere [58, 59]; however, the role of ionizing radiation in inducing diabetes at the lower doses that are the focus of this review remains uncertain, since, based on an early report, no increase has been observed in the AHS [60]. Parathyroid hormone increases with dose in the Japanese atomic bomb survivors, suggesting that there may be radiation-associated hyperparathyroidism [61]. Parathyroid hormone (PTH) has a central role in well-regulated calcium homeostasis and its release is triggered by a decrease in serum calcium levels. Primary hyperparathyroidism results in overproduction of PTH, mobilizing excess calcium to the bloodstream [62]. This elevation results in hypertension (via disturbances in the renin-angiotensin-aldosterone system), cardiac hypertrophy, and myocardial dysfunction [62]. PTH receptors are present in the myocardium and exert hypertrophic effects on cardiomyocytes [62]. These associations suggest plausible mechanisms whereby the elevated PTH concentrations that result from hyperparathyroidism may be involved in various pathological processes that lead to circulatory disease.

## Conclusions

The evidence in this review provides suggestive evidence in support of a causal association between moderate- and low-level radiation exposure and circulatory disease. However, the substantial heterogeneity for certain endpoints limits interpretation of the epidemiological associations.

## Footnotes

1 The equivalent dose, in sievert (Sv), with different types of radiation absorbed dose weighted by their biological effectiveness at inducing stochastic effects, is numerically very close to the (unweighted) absorbed dose, in gray (Gy), for all studies considered here.
